# WU Polyomavirus Infection in Children, Germany

**DOI:** 10.3201/eid0104.071325

**Published:** 2008-04

**Authors:** Florian Neske, Kerstin Blessing, Franziska Ullrich, Anika Pröttel, Hans Wolfgang Kreth, Benedikt Weissbrich

**Affiliations:** * University of Würzburg, Würzburg, Germany; † University Hospital of Würzburg, Würzburg, Germany

**Keywords:** cells, viruses, prions, molecular biology, letter

**To the Editor**: The human polyomaviruses JC and BK are known to cause persisting infections, which are usually asymptomatic in immunocompetent patients but may lead to severe disease in those who are immunosuppressed (*1*). Recently, 2 novel viruses of the family *Polyomaviridae* were detected in respiratory samples and named KI (*2*) and WU polyomavirus (WUPyV) (*3*). To investigate the frequency of WUPyV infections in Germany, we examined nasopharyngeal samples from hospitalized children with acute respiratory diseases for WUPyV DNA.

The samples tested for WUPyV infection consisted of stored nasopharyngeal aspirates (NPA) of hospitalized children at the Children’s Hospital, University of Würzburg. The samples had been received for routine screening of respiratory viruses from January 2002 through September 2005 and from January 2007 through July 2007. All samples were routinely tested for antigens of adenoviruses, influenza viruses A (fluA) and B, parainfluenza viruses 1–3, and respiratory syncytial virus (RSV) by indirect immunofluorescence assays (Chemicon, Temecula, CA, USA). Remaining NPA material was stored at –20°C. DNA was extracted from the samples by using the High Pure Viral Nucleic Acid Kit (Roche, Mannheim, Germany) and stored at –70°C for further testing. All samples were also tested for human bocavirus (hBoV) DNA by PCR (*4*).

WUPyV PCR was performed by using the primer pair AG0048 and AG0049 described by Gaynor et al. (*3*). PCRs were conducted in a 50-μL volume consisting of 5-μL extracted DNA, 1× Qiagen HotStar buffer (QIAGEN, Hilden, Germany), dNTPs at final concentrations of 200 μmol/L each, 200 pmol of each primer, and 1.5 U of HotStarTaq polymerase. The cycling conditions were 50 cycles (94°C for 30 s, 53°C for 40 s, and 72°C for 1 min) after a preheating step of 10 min at 95°C. All PCR products of positive reactions by agarose gel electrophoresis with ethidium bromide staining were sequenced completely in both directions for confirmation of sequence specificity. One negative control was extracted and amplified for every 5 NPA samples. A plasmid containing the cloned PCR product was used as positive control. The sensitivity of the WUPyV PCR was 8.8 copies per reaction as determined by probit analysis, which corresponds to 440 copies per mL of sample. The study was approved by the ethics committee of the medical faculty at the University of Würzburg.

During the study period, 1,326 NPA of hospitalized children with febrile respiratory tract diseases were received for viral diagnostic evaluation. The median age of the patients was 1.6 years (mean age 3.2 years; range 7 days–22 years), and 58.4% were boys. DNA of 1,277 NPA from 1,085 children was available for retrospective testing. Of these, 62 (4.9%) samples from 59 children were positive by WUPyV PCR and subsequent sequencing. The median age of the WUPyV-positive children was 3.0 years (mean 2.9 years; range 4 months–6.3 years) (Figure), and 57% were boys. Of the children with WUPyV-positive NPA, 3.2% were >6 years of age, although children in this age group constituted 15.7% of the total population. Infections with WUPyV were found year round, but most occurred in the winter months. Yearly frequencies (July–June) of WUPyV-positive results varied from 3.2% to 8.5% during the observation period. These variations were not statistically significant. In 34 (54.8%) of the WUPyV-positive samples, co-infections with other respiratory viruses were detected, most frequently with adenovirus (n = 10) and fluA (n = 10), followed by hBoV (n = 9) and RSV (n = 5). The co-infections included 4 triple infections (2 fluA/hBoV/WUPyV, 1 adenovirus/hBoV/WUPyV, and 1 RSV/hBoV/WUPyV). Clinical data were available for 57 of the 62 WUPyV-positive NPA. A broad spectrum of both upper (45.6%) and lower (54.4%) respiratory tract diseases was observed. The latter included bronchitis, wheezing bronchitis, and pneumonia.

**Figure:**
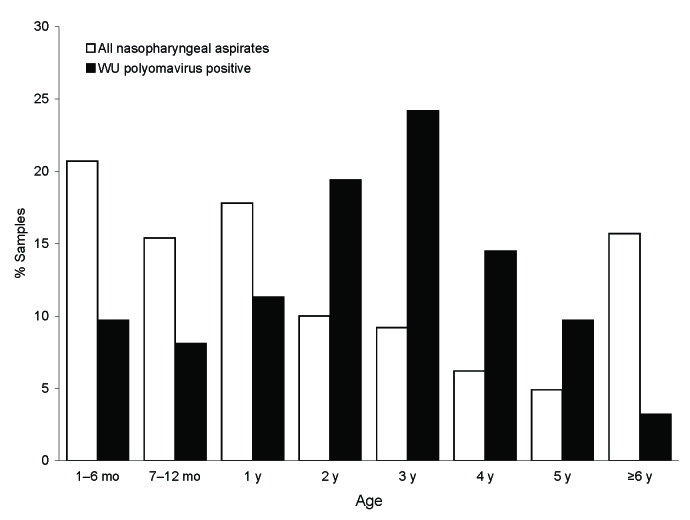
Age distribution of children with WU polyomavirus DNA–positive nasopharyngeal aspirates compared with the age distribution of the total study population.

In the context of the previous reports of WUPyV detection in Australia and North America (*3*), our data suggest a worldwide distribution of WUPyV. Most of the WUPyV-positive children were <4 years of age, and WUPyV DNA was rarely found in children >6 years of age. This age distribution is compatible with WUPyV infection occurring in day nurseries and kindergartens. In keeping with the findings of Gaynor et al. (*3*), we observed a high number of co-infections. The true number of co-infections in our study is probably higher than the reported 53.2% because we did not test for several respiratory pathogens, such as coronaviruses, rhinoviruses, enteroviruses, and the human metapneumovirus. Hypotheses to account for the detection of WUPyV in respiratory samples include the following: WUPyV is a persisting asymptomatic virus that is detected by chance, WUPyV is a persisting virus that is reactivated by an inflammatory process, or WUPyV is a predisposing or aggravating factor of respiratory diseases. Further studies are necessary to determine whether WUPyV is a human pathogen.
